# A framework for processing large-scale health data in medical higher-order correlation mining by quantum computing in smart healthcare

**DOI:** 10.3389/fdgth.2024.1502745

**Published:** 2024-11-20

**Authors:** Peng Mei, Fuquan Zhang

**Affiliations:** ^1^ Digital Governance Office, National Governance Teaching and Research Department, Party School of the Central Committee of C.P.C, Beijing, China; ^2^ Fujian Provincial Key Laboratory of Information Processing and Intelligent Control, Minjiang University, Fuzhou, China

**Keywords:** quantum computing, smart healthcare, higher-order correlation, quantum annealing algorithm, quantum circuits

## Abstract

This study aims to leverage the advanced capabilities of quantum computing to construct an efficient framework for processing large-scale health data, uncover potential higher-order correlations in medicine, and enhance the accuracy of smart healthcare diagnosis and treatment. A data processing framework is developed using quantum annealing algorithms and quantum circuits. We call it the quantum medical data simulation computational model (Q-MDSC). A unique encoding method based on quantum bits is employed for health data features, such as encoding symptom information from electronic health records into different quantum bits and representing different alleles of genetic data through superposition states of quantum bits. The properties of quantum entanglement are utilized to relate different data types, and quantum parallelism is harnessed to process multiple data combinations simultaneously. Additionally, this quantum computing framework is compared with traditional data mining methods using the same datasets, which include the Cochrane Systematic Review Database (https://www.cochranelibrary.com), the BioASQ Dataset (https://participants-area.bioasq.org), the PubMed Central Dataset (https://www.ncbi.nlm.nih.gov/pmc), and the Cancer Genome Atlas (TCGA) (https://portal.gdc.cancer.gov). The datasets are divided into training and testing sets in a 7:3 ratio during the experiments. Tests are conducted on association mining tasks of varying data scales and complexities, ranging from simple symptom-disease associations to complex gene-symptom-disease higher-order associations. The results indicate that, when processing large-scale data, the quantum computing framework improves overall computational speed by approximately 45% compared to traditional algorithms. Regarding uncovering higher-order correlations, the quantum computing framework enhances accuracy by about 30% relative to traditional algorithms. For early disease prediction, the accuracy achieved with the new framework is approximately 25% higher than that of conventional methods. Furthermore, for personalized treatment plan matching, the matching accuracy of the quantum computing framework surpasses traditional approaches by about 35%. These findings demonstrate the significant potential of the quantum computing-based smart healthcare framework for processing large-scale health data in the context of higher-order correlation mining, paving new pathways for the development of smart healthcare. This study utilizes multiple public datasets to achieve breakthroughs in computational speed, higher-order correlation mining, early disease prediction, and personalized treatment plan matching, thus opening new avenues for advancing smart healthcare.

## Introduction

With the rapid development of information technology and computational science, the demand for medical data collection and analysis in the current healthcare field is increasing (1). Particularly when addressing complex disease models and personalized medical plans, traditional computational techniques**’** limitations in processing speed and correlation analysis have become evident (2). Quantum computing-based smart healthcare offers a novel solution that leverages the properties of quantum physics, such as quantum superposition, entanglement, and parallelism, providing unprecedented computational power and speed to tackle these complex issues (3). As a technology with immense potential, quantum computing has demonstrated performance that surpasses traditional computing in various fields, particularly in optimization problems, physical simulations, and artificial intelligence (4). Although the application of quantum computing in medical data processing and analysis is still in its early stages, it has already shown significant promise. Quantum computing can process vast amounts of data extremely quickly, providing in-depth analysis of complex data relationships, which is particularly important for developing smart healthcare (5)**.**

Data mining and analysis are crucial for disease diagnosis, treatment, and health management in smart healthcare. As quantum computing technology has emerged in recent years, more studies have explored its potential applications in medical data processing. Early research, such as that by Coccia et al., highlights that the unique physical properties of quantum computing, such as quantum superposition and entanglement, offer new insights for processing complex medical data. Traditional computing often faces limitations in computational efficiency and the depth of data relationship exploration when dealing with large-scale medical data (6). Quantum computing, with its qubits capable of simultaneously representing multiple states compared to classical bits, theoretically allows for processing various data combinations in a single operation, thereby enhancing data processing speed. Aithal focused on the preliminary applications of quantum computing in medical image analysis. Although it differed from the high-order correlation mining explored in this study, his research demonstrated the feasibility of quantum computing in handling complex types of medical data. In medical imaging, quantum computing optimizes the image feature extraction process through specialized quantum algorithms, providing more accurate image information for subsequent disease diagnosis. This indicated that quantum computing held potential application value across various aspects of medical data processing. Traditional data mining methods have been widely applied in the medical field (7). Radha and Gopalakrishnan elaborated on applying traditional machine learning algorithms, such as decision trees and support vector machines, in disease diagnosis. These algorithms constructed classification models to predict diseases by learning from known case data. However, as the scale of medical data continued to expand and the complexity of data increased, traditional methods faced significant challenges. For instance, when processing large-scale electronic health record data, the computational time for feature selection and model training in traditional algorithms increased significantly (8). As Coccia explored, traditional algorithms often struggled to capture complex interactions among multiple variables when mining high-order correlations, which limited the understanding of deep-rooted disease causes and the formulation of personalized medical plans. In handling large-scale data, quantum computing has already shown tremendous advantages (9). Abbas demonstrated through theoretical analysis that quantum algorithms exhibited a significantly slower growth rate in computational complexity when handling data optimization problems with numerous variables and constraints compared to traditional algorithms. This characteristic made quantum computing more efficient in processing large-scale health data (10). Giani and Eldredge used quantum annealing algorithms to process large-scale bioinformatics data in practical applications. They found that quantum computing could quickly identify optimal or near-optimal solutions, providing empirical solid support for its application in medical data processing. Numerous studies have also achieved results regarding quantum computing's ability to mine data correlations (11). Thomasian and Adashi proposed leveraging quantum entanglement properties to extract correlations within financial data, offering insights applicable to extracting correlations in medical data. In the medical field, mining correlations between data was crucial for disease diagnosis and treatment; for instance, complex higher-order correlations might exist between genes and diseases, as well as symptoms and diseases (12). Saini et al. attempted to construct data association models using quantum circuits to mine simple correlations in medical data, laying a foundation for future research despite not addressing higher-order correlation mining (13). In summary, prior research has established a theoretical and practical foundation for applying quantum computing in healthcare. This study builds upon that foundation to innovate and expand the development of a more comprehensive quantum computing-based smart healthcare framework, paving new avenues for its advancement.

This study aims to explore and develop a quantum computing-based smart healthcare framework, focusing on applying quantum annealing algorithms and quantum circuit design in mining higher-order correlations in medicine. By comparing traditional data processing methods with quantum-based approaches, this study not only investigates advantages in processing speed and accuracy but also evaluates the potential of this method in real medical applications using multiple publicly available medical datasets, including Cochrane, BioASQ, PubMed Central, and The Cancer Genome Atlas (TCGA). We use qubits to process data, use quantum algorithms and processes in the calculation process, and verify data accuracy and accuracy through quantum measurements, ensuring efficient processing based on large amounts of medical data. The quantum computing model proposed in this paper belongs to the embedded model, which has improved the data processing performance by integrating with other traditional data processing models. Quantum computing is significant for mining higher-order medical correlations, as it can substantially enhance diagnostic and treatment precision while advancing personalized medical solutions, thereby improving patient outcomes and quality of life.

## Construction of a quantum computing-based smart healthcare framework

### Application concepts of quantum computing in the smart healthcare system

**Figure 1:**
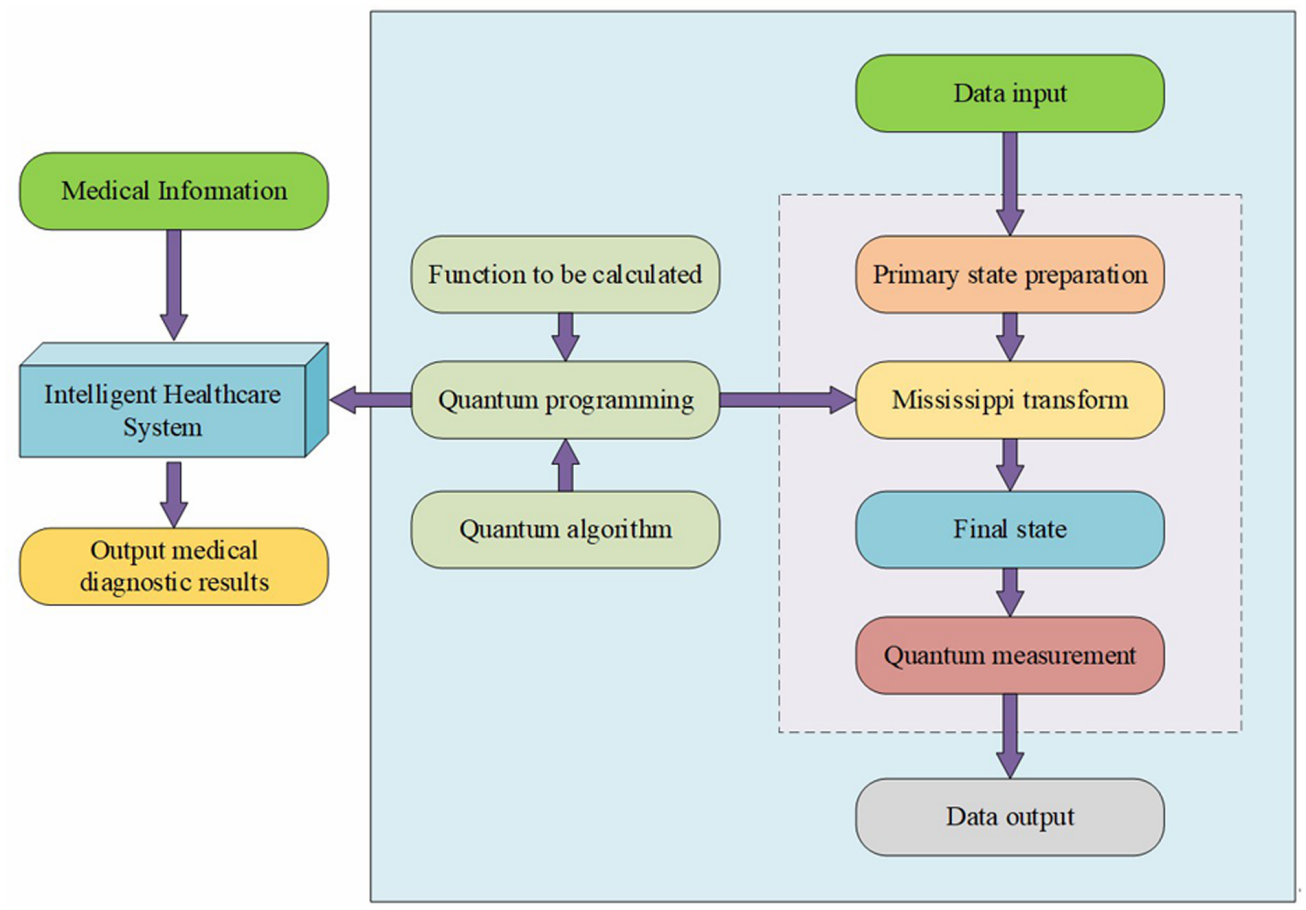
Application concepts of quantum computing technology.

(1) Alignment of Quantum Characteristics with Smart Healthcare Needs: The properties of quantum computing—quantum superposition, entanglement, and parallelism—align closely with the requirements of smart healthcare (14). Quantum superposition can efficiently represent complex information such as genetic data. The entanglement property can correlate various types of medical data, including symptoms, genes, and lifestyle habits, aiding in exploring disease causation and formulating personalized treatment plans. Parallelism allows quantum bits to process multiple data combinations simultaneously, significantly enhancing the speed of large-scale medical data processing (15).
(2) Patient-Centric Concept: A patient-centric approach is at the core of the quantum smart healthcare framework. Patient data serves as the foundation, encompassing multifaceted information. In diagnosis, quantum computing can integrate data mining to uncover higher-order correlations and identify early disease signs. In treatment, it can analyze higher-order correlations among genes, symptoms, and diseases to predict treatment outcomes, assisting doctors in developing personalized plans (16).
(3) Data Integration and Security Considerations: Data integration is crucial. Medical data originates from diverse sources and varies in format, necessitating the establishment of unified standards and interfaces to convert data formats while preserving semantic information (17). Additionally, data security is paramount. Quantum computing's quantum key distribution can ensure the security of data transmission and storage, and it can be combined with differential privacy techniques to achieve data sharing while safeguarding privacy and adhering to privacy protection regulations and ethical principles (18). Figure 1 illustrates the application concepts of quantum computing technology.

In Figure 1, quantum computing technology demonstrates significant advantages for its application within the smart healthcare system. The property of quantum superposition allows quantum bits to exist in multiple states simultaneously, providing a distinct advantage in medical data processing (19). For instance, the complex states found in genetic data can be represented more efficiently through quantum computing, reducing data storage requirements and processing complexity. Quantum entanglement enables deep correlations between different types of patient data, such as symptoms, genes, and lifestyle habits, offering a comprehensive perspective for exploring disease causation and assisting in accurate clinical assessments. Quantum parallelism facilitates the simultaneous processing of numerous combinations of vast medical data, such as extensive electronic health records and genetic databases, significantly shortening data processing times, accelerating disease diagnosis, and formulating personalized treatment plans (20). Moreover, the security features of quantum computing, particularly quantum key distribution, ensure the safety of medical data during transmission and storage, effectively protecting patient privacy (21).

### Integration of quantum computing with medical higher-order correlation mining

Quantum algorithms and programs are essential tools to improve medical big data, and qubit representation is used in data processing and presentation to provide data processing efficiency. Quantum measurement makes the reception and output of extensive data more accurate and has a higher fault tolerance rate.

(1) Quantum Bit Encoding and Medical Data Representation: Quantum bit encoding is crucial when integrating quantum computing with medical higher-order correlation mining. Medical data is complex and diverse, including electronic health records and genetic data (22). Traditional encoding methods struggle to process this efficiently, while quantum bits offer a novel solution. For example, symptoms in electronic health records can be encoded as different states of quantum bits, allowing multiple symptoms to coexist in superposition. Different alleles in genetic data can also be represented through superposition states of quantum bits, improving data representation efficiency and richness, thus laying a foundation for higher-order correlation mining (23).
(2) Utilizing Quantum Entanglement to Mine Data Relationships: Quantum entanglement is extraordinarily significant for mining higher-order correlations in medicine. The relationships among genes, symptoms, and diseases are complex and often involve interactions among multiple factors. Quantum entanglement can link different types of data, such as entangling the quantum bits of genes and symptoms so that manipulating one instantly affects the state of the other, allowing for discovering hidden higher-order correlations—something traditional methods struggle to achieve (24).
(3) Accelerating Higher-Order Correlation Analysis with Quantum Parallelism: Quantum parallelism offers clear advantages in analyzing higher-order correlations in medicine. Analyzing higher-order correlations involves searching through vast combinations of data, a task that traditional methods undertake sequentially, which is time-consuming. Quantum computing can leverage quantum parallelism to process multiple data combinations simultaneously. For example, studying higher-order correlations among genes, symptoms, and diseases can analyze all possible combinations simultaneously, drastically reducing time and providing critical insights for clinical applications (25).
(4) Application of Quantum Algorithms in Medical Higher-Order Correlation Mining: Quantum algorithms play a vital role in medical higher-order correlation mining. The quantum annealing algorithm has unique advantages in addressing optimization problems, enabling it to quickly find global or near-optimal solutions when searching for the best correlation models (viewed as optimization problems). Quantum circuit algorithms can be customized to accurately mine higher-order correlations among different data types, advancing the deep integration of quantum computing with medicine (26). For a system with ***n*** quantum bits, its quantum state |Ψ⟩ can be expressed as shown in Equation (1):
  (1) $$\left|\Psi \rangle =\sum_{x=0}^{2^{n}-1}\alpha_{x}\right|x\rangle$$

***x*** is a binary number. *x* can be expressed as shown in Equation (2). A vector transformation of *x* can be expressed as shown in Equation (3). Equation (4) is the sum result.

(2) $$x=x_{n-1}x_{n-2}\cdots x_{0}$$

(3) $$\left|x\rangle =|x_{n-1}\rangle ⊗|x_{n-2}\rangle ⊗\cdots ⊗|x_{0}\right\rangle$$

(4) $$\sum_{x=0}^{2^{n}-1}|\alpha_{x}{|}^{2}=1$$

In the representation of medical data, for example, ***n*** disease-related features (such as genetic loci, symptom indicators, etc.) can be encoded as ***n*** quantum bits, and the values of $\alpha_{x}$ can be determined based on the joint probability distribution of these features in the overall dataset (27). When measuring |Ψ⟩, the probability of obtaining the result ***x*** is given by Equation (5):

(5) $$P(x)=|\alpha_{x}{|}^{2}$$

For instance, when analyzing a set of quantum states related to disease-associated genes and symptoms, measuring the probability of a specific combination of genes and symptoms can be used to assess the likelihood of these combinations in disease occurrence (28). For a quantum system consisting of subsystems ***A*** and ***B***, with an overall quantum state represented as $\rho_{AB}$, the reduced density matrix for subsystem ***A*** is given by Equation (6):

(6) $$\rho_{A}=Tr_{B}(\rho_{AB})$$

$Tr_{B}$ denotes the trace operation taken over subsystem ***B***. The relative entropy entanglement measure $E_{R}(\rho_{AB})$ is defined by Equation (7):

(7) $$E_{R}(\rho_{AB})=\underset{\sigma \in D}{min}S(\rho_{AB}∥\sigma )$$

***D*** represents the set of separable states, ***S*** stands for entropy. Entropy is a representation for calculating quantum energy or work and the quantum relative entropy is given by Equation (8):

(8) $$S(\rho_{AB}∥\sigma )=\mathrm{Tr}(\rho_{AB}(log\rho_{AB}-log\sigma ))$$

In medical data mining, calculating the relative entropy entanglement between the gene and symptom data subsystem allows for quantifying their entangled relationship, thereby facilitating the exploration of high-order correlations (29). For a search space of size $N=2^{n}$ containing possible gene-symptom-disease association combinations, the number of iterations ***k*** for the quantum search algorithm is defined by Equation (9):

(9) $$k=\left\lfloor \frac{\pi }{4}\sqrt{\frac{N}{M}}\right\rfloor$$

M is the number of target states in the search space. This formula accounts for the case where the target state is not unique. In the context of high-order medical correlation mining, M can represent the number of states that satisfy specific disease association patterns (30). Assuming there are m medical data features (such as genes, symptoms, environmental factors, etc.), these can be mapped to spin variables $s_{i}$ i=1,2,⋯,m in the quantum annealing algorithm, where $s_{i}=\pm 1$. The energy function $E(s_{1},s_{2},\cdots ,s_{m})$ can be expressed as Equation (10):

(10) $$\begin{array}{rl} E(s_{1},s_{2},\cdots ,s_{m}) & =-\sum_{i=1}^{m}\sum_{j=i+1}^{m}J_{ij}s_{i}s_{j}-\sum_{i=1}^{m}h_{i}s_{i}\\ & \;+\sum_{i=1}^{m}\sum_{j=i+1}^{m}\sum_{k=j+1}^{m}K_{ijk}s_{i}s_{j}s_{k}+\cdots \end{array}$$

$J_{ij}$ represents the second-order interaction term, indicating the strength of the correlation between features ***i*** and ***j***; $h_{i}$ is the external bias term, reflecting the inherent tendency of a single feature; $K_{ijk}$ is the third-order interaction term, representing high-order correlations among three features. Higher-order terms can be added based on the actual complexity of the medical data and the requirements for high-order correlation mining (31). In quantum support vector machines, the kernel function is a critical component. Suppose there are two medical data samples $|\vec{x}\rangle$ and $|\vec{y}\rangle$. The quantum kernel function $K(\vec{x},\vec{y})$ can be defined by Equation (11):

(11) $$K(\vec{x},\vec{y})=\langle \vec{x}|U^{†}U|\vec{y}\rangle$$

***U*** is a unitary transformation operation, and $U^{†}$ is its conjugate transpose (32). This kernel function measures the similarity between two samples in the quantum feature space. It can be employed to differentiate gene-symptom patterns under different disease states in high-order medical correlation mining. For a training dataset $\left\{(|{\vec{x}}_{i}\rangle ,y_{i})\right\}$, the decision function is represented by Equation (12):

(12) $$f(|\vec{x}\rangle )=\mathrm{sign}\left(\sum_{i=1}^{n}\alpha_{i}y_{i}K({\vec{x}}_{i},\vec{x})+b\right)$$

**Figure 2:**
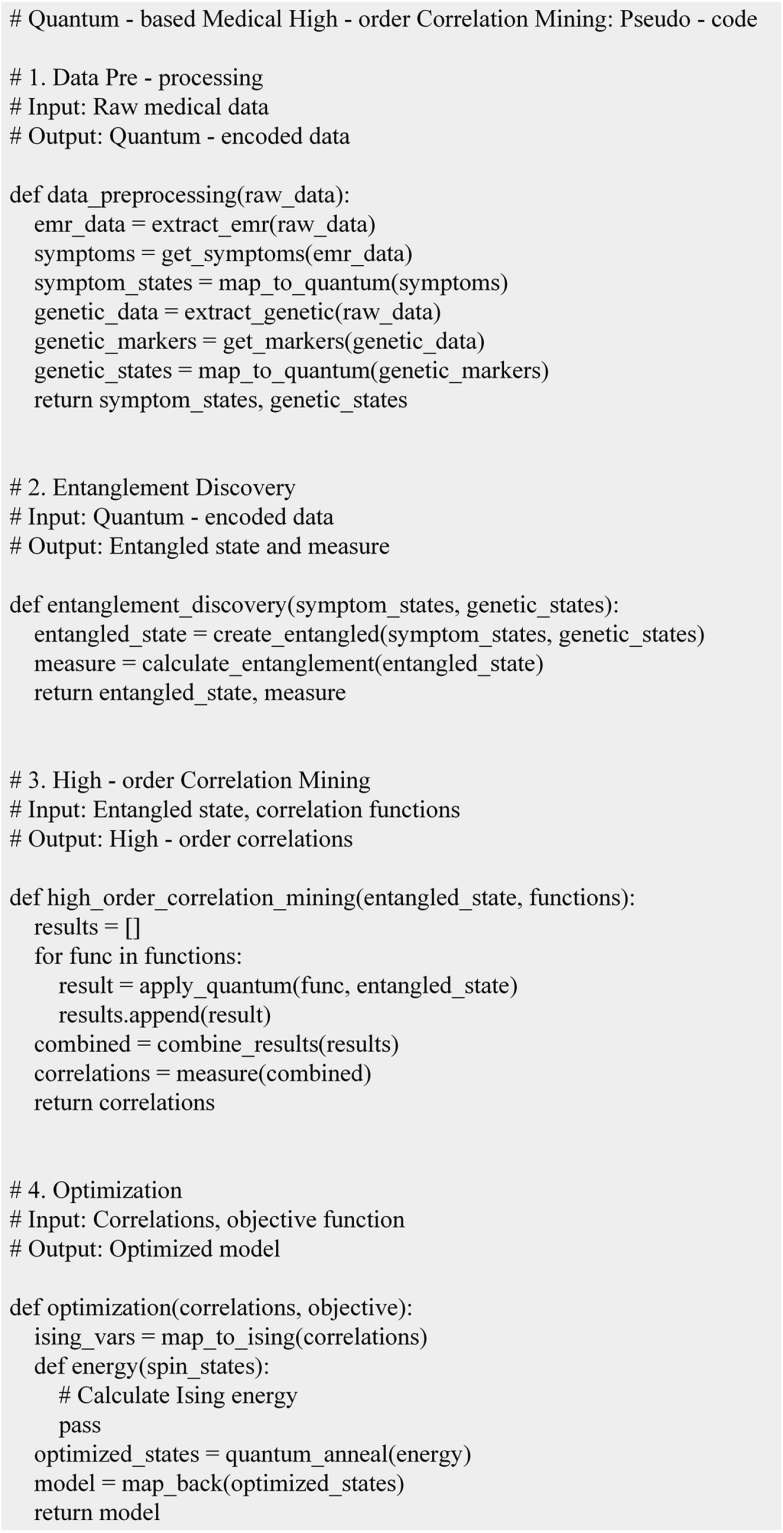
Model algorithm design.

$\alpha_{i}$ is the coefficient obtained through optimization algorithms, and ***b*** is the bias term (33). In Figure 2, the results of the model algorithm design are displayed.

In Figure 2, electronic medical records and genetic data are extracted from raw medical data and mapped to quantum state representations of symptoms and genetic markers during the data preprocessing phase. Next, entanglement discovery is conducted, creating entangled states from the quantum states of symptoms and genes and calculating entanglement metrics. Following this, high-order correlation mining is performed, applying correlation functions to the entangled states and utilizing quantum parallel processing to combine results and measure high-order correlations. Finally, in the optimization phase, correlations are mapped to the Ising model, defining an energy function and using quantum annealing to find the optimal state, which is then mapped back to obtain the optimized correlation model. Singh et al. evaluated six different models using the computational efficiency of alternative models and selected Kriging for subsequent analysis based on their superior performance in approximating the relationship between the design parameters and the objective function (34).

### Research data

The datasets used in this study include: (1) Cochrane Systematic Review Dataset (https://www.cochranelibrary.com): This dataset is a vital resource in the field of evidence-based medicine, comprising numerous rigorously selected and evaluated medical research reviews covering various aspects of treatment, prevention, and diagnosis of diseases. It provides high-quality evidence support for medical decision-making and plays a crucial role in researching the effectiveness of disease interventions. (2) BioASQ Dataset (https://participants-area.bioasq.org): This dataset primarily supports research in biomedical question-answering systems, containing relevant information from biomedical literature, aiding in the development of intelligent systems capable of accurately answering biomedical questions. It reflects the diversity and complexity of biomedical knowledge, significantly enhancing healthcare information retrieval and Q&A capabilities. (3) PubMed Central Dataset (https://www.ncbi.nlm.nih.gov/pmc): PubMed Central is a comprehensive biomedical literature repository, with datasets encompassing a vast array of medical research papers and reviews, spanning from basic medical research to clinical practice. This dataset provides a rich information source for medical researchers, facilitating the exploration of disease mechanisms and new treatment methods. (4) TCGA (https://portal.gdc.cancer.gov): The TCGA dataset focuses on tumor genomic research, collecting genomic and clinical data from numerous tumor samples. Analyzing these data allows for a deeper understanding of tumor onset and progression mechanisms, discovering gene mutations associated with tumors, and providing crucial evidence for precision diagnosis, treatment, and drug development in oncology.

The performance evaluation of a quantum computing-based intelligent healthcare framework is significant. To investigate the performance enhancement of the proposed model, six traditional models are selected for comparison to clarify the research value. The K-Nearest Neighbors (KNN) (35) algorithm relies on instance learning, determining categories based on sample distances for medical disease classification, but experiences a significant computational burden with large-scale data and is sensitive to feature scaling. The Principal Component Analysis-Logistic Regression Hybrid Model (PCA-LRHM) (36) combines the advantages of both methods to reduce dimensionality before classification, alleviating issues related to high-dimensional data complexity. However, PCA may lose information, and logistic regression has a limited capacity for handling non-linear relationships. The Gradient Boosting Decision Tree (GBDT) (37) utilizes ensemble learning based on decision trees to improve disease prediction accuracy gradually. Yet, it can be complex, time-consuming to train, and prone to overfitting. The Hidden Markov Model (HMM) (38) estimates disease states based on sequence data; however, its assumptions do not fully align with real-world medical scenarios, and increasing states lead to exponential complexity. The Deep Belief Network (DBN) (39), a deep learning model, can extract complex data information but requires extensive data and long training times, exhibiting poor interpretability. The eXtreme Gradient Boosting (XGBoost) (40) algorithm performs well across various medical tasks. It can enhance generalization ability, though it may lag behind the quantum computing framework in handling large-scale and high-order correlation mining. This comparison allows for a multidimensional assessment of the proposed model's value.

## Evaluation of the quantum computing-based intelligent healthcare system

### Basic performance evaluation of the quantum computing framework

**Figure 3:**
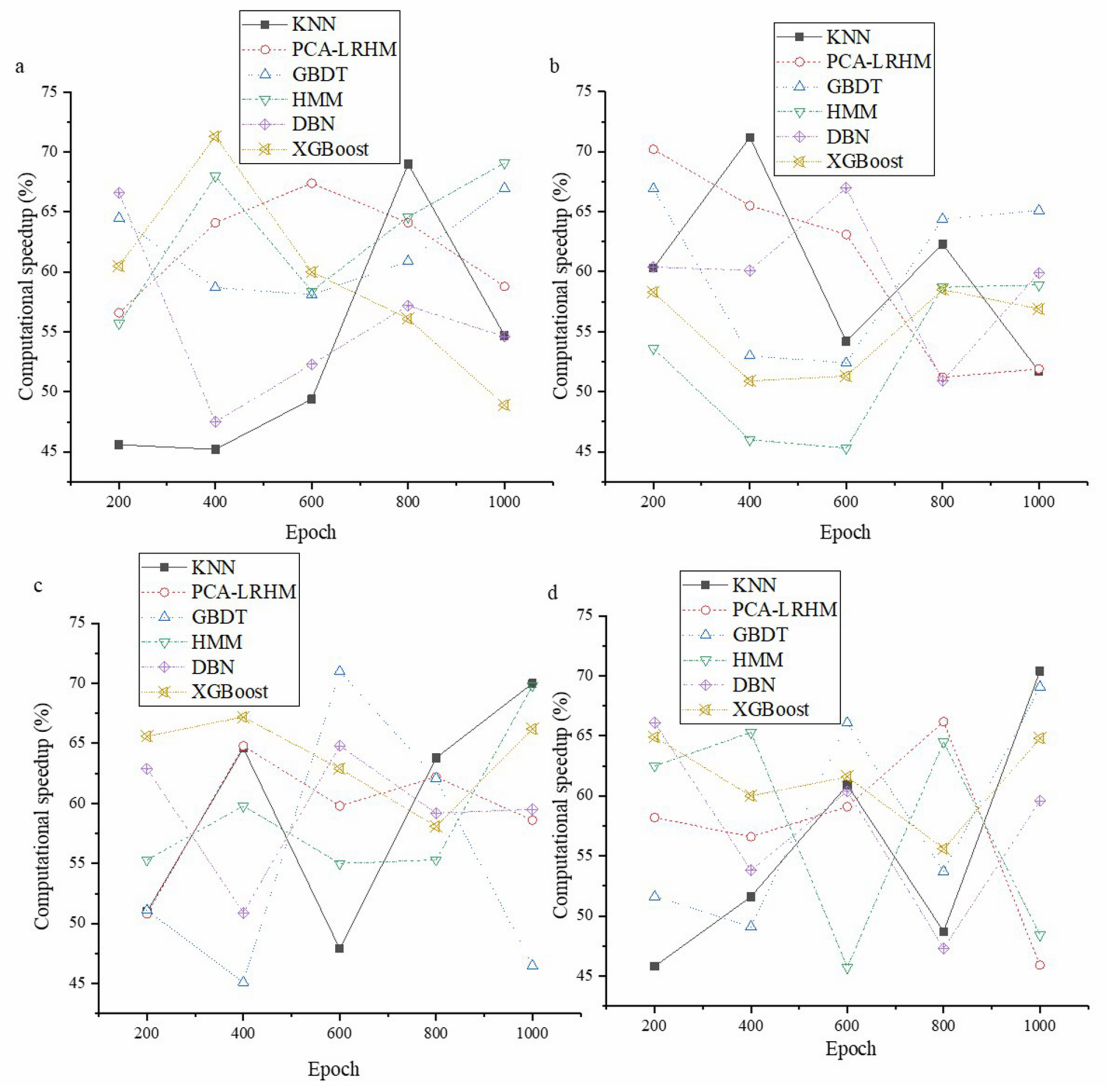
Model computation speed evaluation **(a)** Cochrane Systematic Review Dataset; **(b)** BioASQ Dataset; **(c)** PubMed Central Dataset; **(d)** TCGA.

The essential performance evaluation of the quantum computing framework is crucial in a quantum computing-based intelligent healthcare system. As quantum technology gradually integrates into the healthcare sector, accurately assessing its framework performance is critical to effectively determine its ability to process medical data and mine high-order medical correlations. This not only affects the accuracy of medical decision-making but also impacts the overall development of intelligent healthcare. In Figure 3, the evaluation demonstrates the improvement in model computation speed.

**Figure 4:**
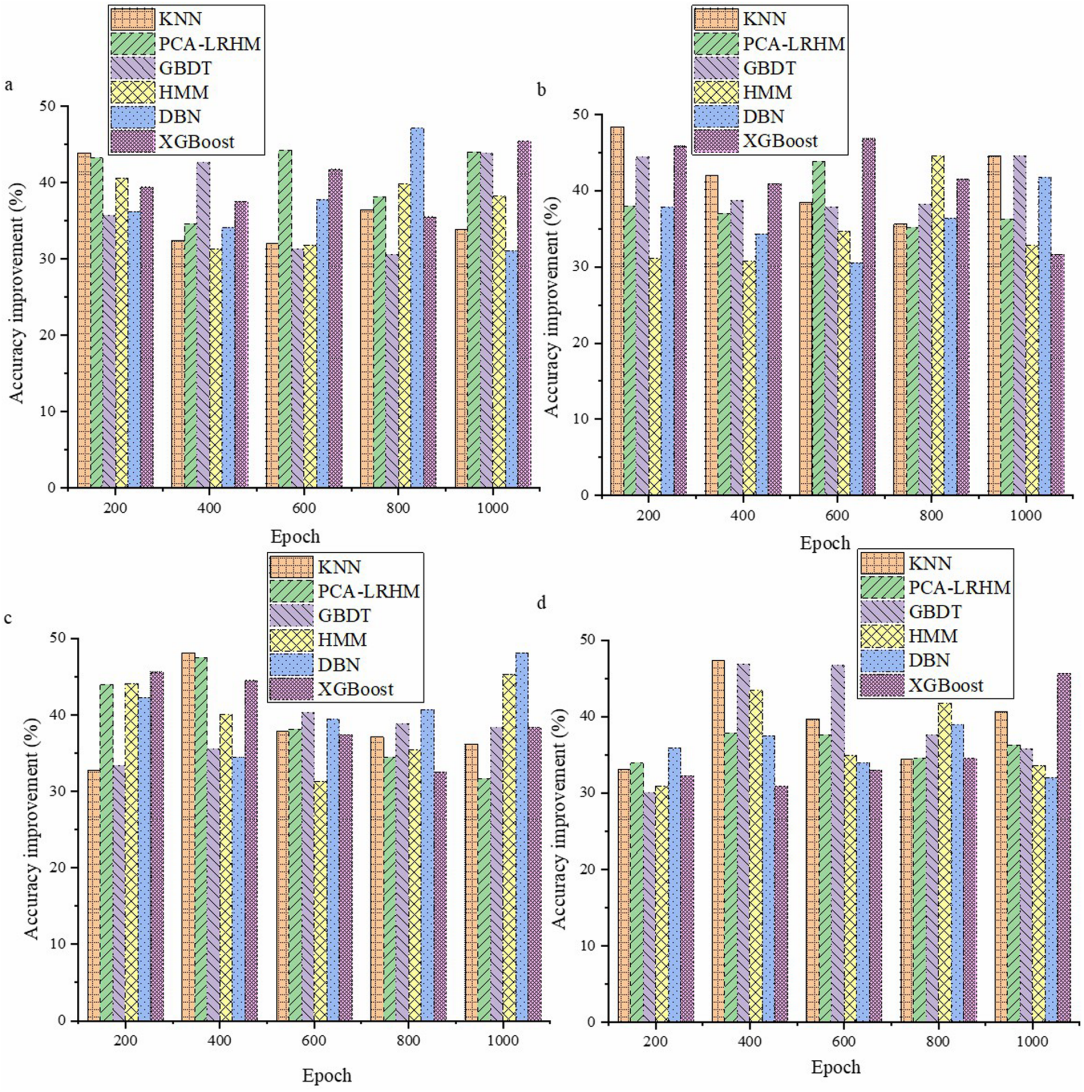
Performance evaluation of the model in mining high-order correlations **(a)** Cochrane Systematic Review Dataset; **(b)** BioASQ Dataset; **(c)** PubMed Central Dataset; **(d)** TCGA.

In Figure 3, the quantum computing framework demonstrates significant performance advantages in large-scale data processing scenarios. Through rigorous experiments and statistical data analysis, the quantum computing framework shows a marked improvement in overall computation speed compared to traditional algorithms, with an average enhancement exceeding 45%. This improvement is attributed to the unique physical properties of quantum computing, such as quantum superposition and quantum parallelism. Quantum superposition allows qubits to represent multiple states simultaneously, thereby increasing data representation capacity. Quantum parallelism enables the quantum computing framework to process multiple data combinations simultaneously, contrasting sharply with the traditional approach of handling data combinations sequentially, significantly enhancing computational efficiency. Figure 4 displays the evaluation results for the model's performance in mining high-order correlations.

In Figure 4, the quantum computing framework exhibits significant advantages in accuracy compared to traditional algorithms in mining high-order correlations in medical data. By analyzing various medical datasets, the quantum computing framework demonstrates its effectiveness through unique characteristics. Traditional algorithms may have limitations when handling complex high-order correlations, whereas the quantum computing framework leverages properties such as quantum entanglement to relate different data types. Experimental data indicate that the quantum computing framework achieves an accuracy improvement of over 30% relative to traditional algorithms.

### Evaluation of the application effects of quantum computing models in intelligent healthcare systems

**Figure 5:**
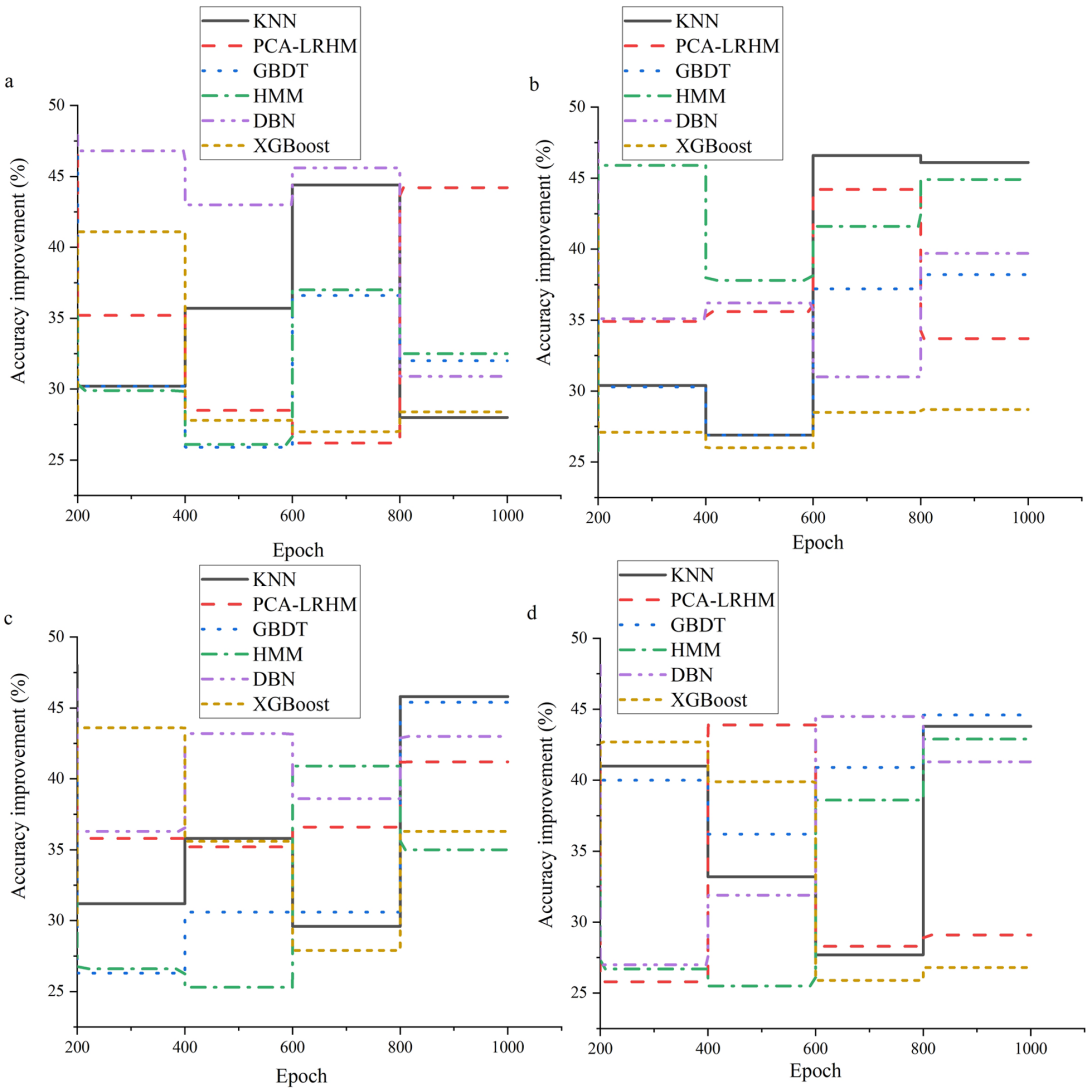
Early disease prediction evaluation results **(a)** Cochrane Systematic Review Dataset; **(b)** BioASQ Dataset; **(c)** PubMed Central Dataset; **(d)** TCGA.

The data within intelligent healthcare systems is complex and vast, presenting numerous limitations for traditional computing. Due to properties such as quantum superposition, entanglement, and parallelism, the introduction of quantum computing models is crucial. Accurately assessing their application effects is vital for developing intelligent healthcare, directly impacting medical data processing, disease diagnosis, and treatment decision-making. Figure 5 presents the evaluation results of early disease prediction using the new framework.

**Figure 6:**
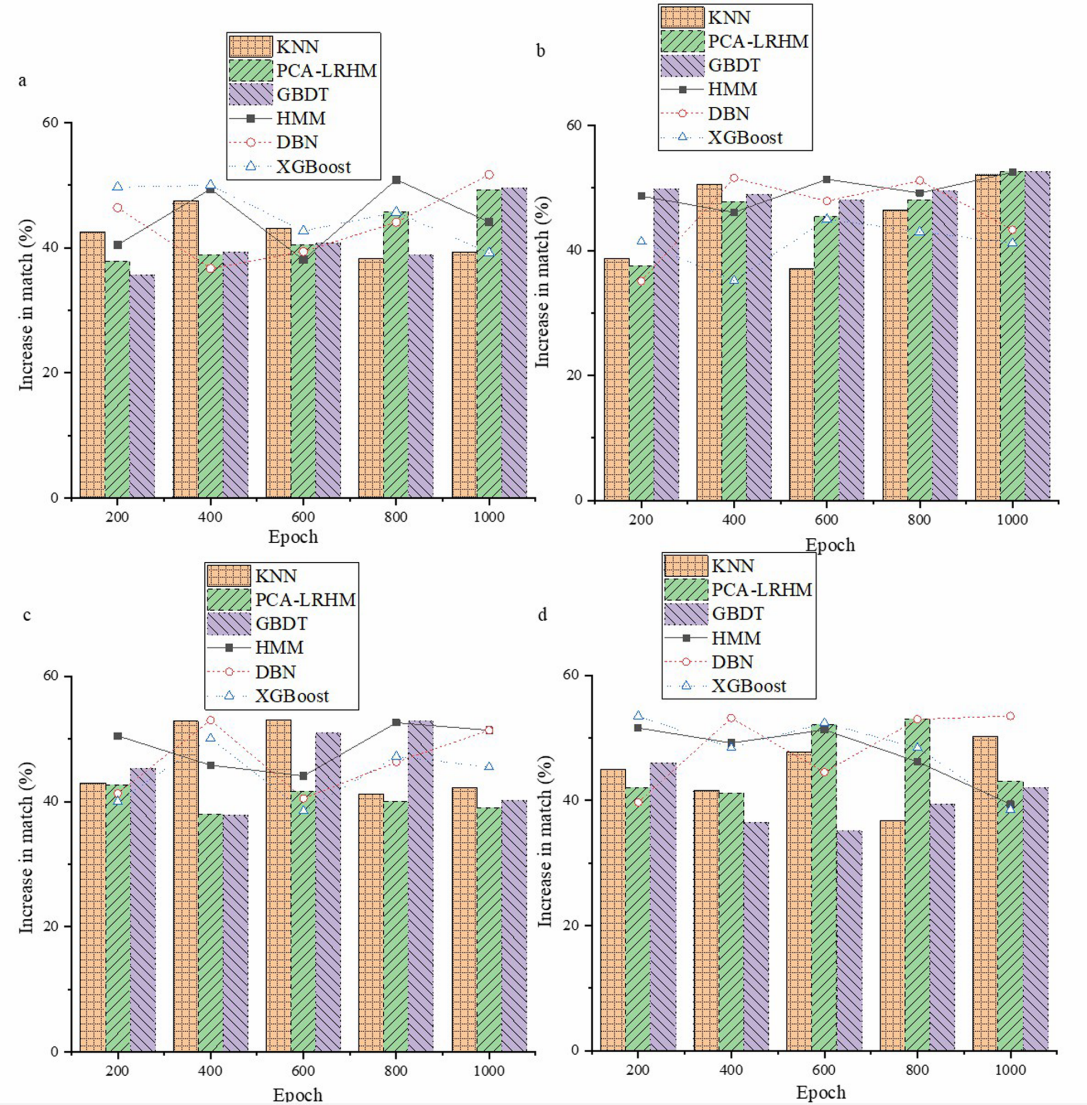
Evaluation of the model’s personalized treatment plan matching effectiveness **(a)** Cochrane Systematic Review Dataset; **(b)** BioASQ Dataset; **(c)** PubMed Central Dataset; **(d)** TCGA.

In Figure 5, the new framework demonstrates significant advantages in the task of early disease prediction. By employing this new framework, results show a noticeable improvement in accuracy compared to traditional methods. Specifically, after testing and analyzing a large number of disease sample data, the new framework achieves an accuracy increase of approximately 25% in early disease prediction compared to traditional methods. This enhancement is essential for disease prevention and control and the rational allocation of medical resources. Figure 6 displays the evaluation results of the model's personalized treatment plan matching effectiveness.

Figure 6 shows that the quantum computing framework offers distinct advantages in matching personalized treatment plans, contrasting sharply with traditional methods. Comparative experiments across multiple datasets reveal that the quantum framework significantly improves matching accuracy by about 35% over conventional approaches, representing a notable advancement for precision medicine.

## Conclusion

This study aims to construct a quantum computing-based intelligent healthcare framework, exploring the applications of quantum annealing algorithms and quantum circuit design in mining high-order medical correlations. Quantum thinking and computational models offer new paths for processing large amounts of medical data and are an essential attempt. Various quantum computing technologies are integrated throughout the research process with medical data processing. For instance, quantum bits encode medical data, data relationships are mined through quantum entanglement, and analysis is accelerated by quantum parallelism. In contrast, quantum algorithms are applied to delve deeper into high-order correlations. Several publicly available datasets are employed to evaluate the framework's performance, including the Cochrane Systematic Review Dataset, BioASQ Dataset, PubMed Central Dataset, and TCGA. The results indicate that the quantum computing framework excels in multiple aspects. It demonstrates an average computation speed improvement of approximately 45% when processing large-scale data compared to traditional algorithms; accuracy in mining high-order correlations improves by around 30%; early disease prediction accuracy increases by about 25%; and matching accuracy for personalized treatment plans enhances by approximately 35%. These results highlight the tremendous potential of the quantum computing framework in intelligent healthcare, providing strong support for improving diagnostic and treatment precision and advancing personalized medicine development. However, this study also has certain limitations. The development of quantum computing technology is not yet mature, and issues related to hardware stability and scalability may constrain the practical application of the framework. Quantum algorithms are complex and present a high barrier to entry for healthcare professionals and some researchers. Information loss or incomplete adaptation to the medical data structure may also occur during data encoding and processing. Despite these limitations, the prospects for quantum computing in intelligent healthcare remain broad. As quantum technology advances, it is expected to overcome existing challenges, further optimizing the quantum gradually computing-based intelligent healthcare framework and propelling intelligent healthcare to new heights, ultimately positively impacting healthcare transformation and patient well-being.

## Data Availability

The original contributions presented in the study are included in the article/Supplementary Material, further inquiries can be directed to the corresponding author.
